# Proposed Measurement of the Beta-Neutrino Correlation in Neutron Decay

**DOI:** 10.6028/jres.110.060

**Published:** 2005-08-01

**Authors:** B. Collett, R. Anderman, S. Balashov, F. B. Bateman, J. Byrne, M. S. Dewey, B. M. Fisher, L. Goldin, G. Jones, A. Komives, T. Konopka, M. Leuschner, Yu. Mostovoy, J. S. Nico, A. K. Thompson, C. Trull, F. E. Wietfeldt, R. Wilson, B. G. Yerozolimsky

**Affiliations:** Physics Department, Hamilton College, Clinton, NY 13323; Kurchatov Institute, Moscow, Russia; National Institute of Standards and Technology, Gaithersburg, MD 20899; University of Sussex, United Kingdom; National Institute of Standards and Technology, Gaithersburg, MD 20899; Department of Physics, Tulane University, New Orleans, LA 70118; Physics Department, Harvard University, Cambridge, MA 02139; Physics Department, Hamilton College, Clinton, NY 13323; Physics Department, DePauw University, Greencastle, IN 46135; Physics Department, Hamilton College, Clinton, NY 13323; Indiana University Cyclotron Facility, Bloomington, IN 47408; Kurchatov Institute, Moscow, Russia; National Institute of Standards and Technology, Gaithersburg, MD 20899; Department of Physics, Tulane University, New Orleans, LA 70118; Department of Physics, Tulane University, New Orleans, LA 70118; Physics Department, Harvard University, Cambridge, MA 02139

**Keywords:** beta decay, beta-neutrino correlation, neutron decay, CKM unitarit

## Abstract

Currently, the beta-neutrino asymmetry has the largest uncertainty (4 %) of the neutron decay angular correlations. Without requiring polarimetry this decay parameter can be used to measure *λ* (*g_a_/g_v_*), test Cabibbo-Kobayashi-Maskawa (CKM) unitarity limit scalar and tensor currents, and search for Charged Vector Current (CVC) violation. We propose to measure the beta-neutrino asymmetry coeffcient, *a*, using time-of-flight for the recoil protons. We hope to achieve a systematic uncertainty of *σ_a_ / a ≈* 1.0 %. After tests at Indiana University’s Low Energy Neutron Source (LENS), the apparatus will be moved to the National Institute of Standards and Technology (NIST) where the measurement can achieve a statistical uncertainty of 1 % to 2 % in about 200 beam days.

## 1. Introduction and Discussion

The simplicity of the decay of the free neutron, $n\to p+e+{\bar{v}}_{e}+0.783$ Mev, makes it an ideal testing testing ground for precise measurements of the parameters of the Standard Electroweak Model. The most important features of neutron decay are described by the formula of Jackson, Treiman, and Wyld [1], which gives the neutron decay probability as a function of the emitted (***p***_e_) and antineutrino (***p***_ν_) momenta, and the direction of the initial neutron’s spin vector (***σ***):

$$N\propto \frac{1}{\tau_{n}}E_{e}\left|p_{e}\right|{\left(Q-E_{e}\right)}^{2}\left[1+a\frac{p_{e}\cdot p_{\nu }}{E_{e}E_{\nu }}+\sigma \cdot \left(A\frac{p_{e}}{E_{e}}+B\frac{p_{\nu }}{E_{\nu }}+D\frac{p_{e}\times p_{\nu }}{E_{e}E_{v}}\right)\right].$$ (1)

Here *τ*_n_ is the neutron decay lifetime, *E*_e_ and *E*_ν_ are the electron and antineutrino energies, and *Q* is the neutron-proton mass difference: 1293 keV.

The experimental asymmety coeffcients, *a*, *A*, **B**, and *D* are related to the underlying vector (*g*_V_) and axial vector (*g*_A_) coupling constants so that, under reasonable assumptions, a measurement of *τ*_n_ plus any one of *a*, *A*, or *B* determines *g*_A_ and *g*_V_ uniquely. Additional measurements overconstrain the system and test the self-consistency of the Standard Electroweak Model. New physical forces or phenomena can change the relationships between *τ*_n_, *a*, *A*, and *B* slightly, and these could be detected by sufficiently precise experiments.

We propose a new experiment to measure a to a precision of approximately 1 %. This will lead to an improved determination of the *g*_V_ and *g*_A_ coupling constants, improved tests of the Standard Model, limits on new physics, such as second-class currents (see Ref. [2]), and an improved test of the unitarity of the Cabibbo-Kobayashi-Maskawa (CKM) matrix using the neutron decay system.

## 2. Proposed Experimental Method

The currently recommended value for *a* is based on the work of Stratowa et al. (1978) [3] and Byrne et al. (2002) [4] who each measured the shape of the proton spectrum to determine *a* to a fractional uncertainty of 5 %. We propose a new method, based on a proposal of Yerozolimsky and Mostovoy [5–7], which constructs an asymmetry that directly yields *a* without requiring precise proton spectroscopy. The reduction in systematic uncertainties could lead to a five-fold improvement in the precision of *a* at the cost of reduced count rate. This paper describes the experimental system and briefly discusses the potential sources of systematic uncertainties. Considerably more detail can be found in a forthcoming paper by Wietfeldt et. al. [8].

**Fig. 1:**
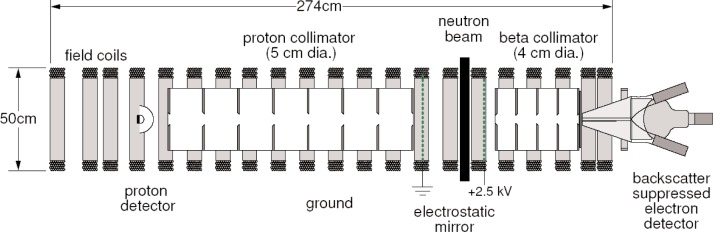
Sketch of the proposed instrument to measure *a*.

The proposed apparatus is shown in Fig. 1. A proton detector and electron detector are positioned on either side of a cold neutron beam. A long solenoid provides a uniform magnetic field, ***B***, aligned to the axis of the detectors. This field transports the electron and proton to the detectors allowing coincidence detection of the electron and proton. Within the solenoid are a series of precisely aligned circular apertures of radius *r*. A proton’s trajectory inside the solenoid is helical, with radius *R* proportional to its transverse momentum *p*_⊥_: R = *p*_⊥_*c/eB* [9]. Only those decay protons with transverse momentum below a threshold value (which depends on the position of the decay vertex) can pass unobstructed through the solenoid and be detected. Similarly, a second set of collimation apertures constrains the transverse electron momentum. A pair of fine wire grids produces an axial electric field in the decay region, directing all decay protons toward the proton detector regardless of their initial axial momenta. Decay electrons are energetic enough to easily pass through this electric field.

**Fig. 2:**
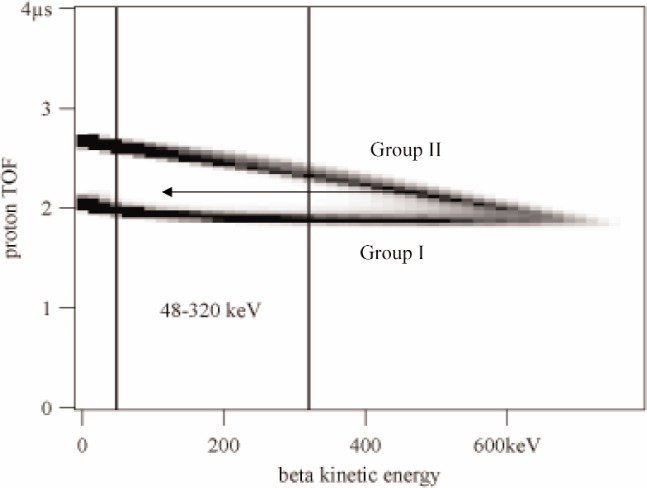
Time-of-flight vs electron energy from a Monte Carlo simulation. For beta kinetic energies less than 320 keV the two proton groups are well-separated. The arrow represents a backscat-tered electron.

Our method relies on converting the initial distribution of angles between the electron and proton into a distribution of times-of-flight for the protons. The a parameter is related to an asymmetry between the number of neutrinos created with axial momenta parallel to the electron momentum and the number created antiparallel. Momentum conservation couples this asymmetry in neutrino populations to an asymmetry in proton populations. The electric field in the region where the neutrons decay sweeps both populations of protons, those with initial axial momenta anti-parallel to the electron axis (group I) and those parallel (group II), toward the proton detector. However the protons in group II must turn around and will take longer to reach the proton detector than those in group I. The time-offlight difference depends on the initial kinetic energy of the protons and thus on the electron energy as seen in Fig. 2. Since the geometry is known precisely, the experimental difference between the number of events in group I (*N*_I_) and the number in group II (*N*_II_) leads directly to *a* value for the a coeffcient:

$$a(E)=\frac{c}{\upsilon_{e}}\left[\frac{2X(E)}{\left(\phi^{I}(E)-\phi^{\text{II}}(E))-X(E)(\phi^{I}(E)+\phi^{\text{II}}(E)\right)}\right].$$ (2)

Here *X*(*E*) is the experimental asymmetry for some slice of electron energy *E*:

$$X(E)=\frac{N_{I}-N_{\text{II}}}{N_{I}+N_{\text{II}}},$$ (3)

*υ*_e_ is the beta electron velocity, and *ϕ*^I^(*E*) and *ϕ*^II^(*E*_e_) are geometrical factors [8]. Although *a* must be measured as a function of *E*, the correlation coefficient is independent of *E* at the proposed level of accuracy.

The experiment will be constructed and undergo initial testing at the Low Energy Neutron Source at the Indiana University Cyclotron Facility (LENS) where ample beam time for this task will be available and will be moved to the National Institute of Standards and Technology (NIST) for actual data collection. Monte-Carlo simulations have shown that an experimental run at NIST of about 200 beam days (a typical run for a neutron decay experiment) will yield a measurement of *a* with a statistical precision of 1.5 %. The precision can be reduced below 1 % either by additional runs at NIST or by moving the experiment to a higher flux cold neutron source such as the Spallation Neutron Source at Oak Ridge (under construction) or the ILL reactor in Grenoble, France. We have designed the method to be systematically limited at the 1.0 % level, a factor of five improvement over previous experiments.

## 3. Systematic Effects

A number of important systematic effects are anticipated. We have taken care to control all expected systematic uncertainties to less than 0.5 % of *a* and will discuss some of the most important here.

### 3.1 Backscatter From the Electron Detector

There is some probability for an electron to back-scatter out of a beta detector without depositing its full energy. Reducing the measured electron energy may move an electron from group II to group I, as shown by the arrow in Fig. 2, producing a false asymmetry. Vetoing these events removes the bias because it affects protons in groups I and II equally.

We will surround the plastic-scintillator detector with veto-detector paddles to reject electrons that are backscattered from the primary detector. The magnetic field at the energy detector is low, so if the electron backscatters it has little chance to be transported back through the entrance of the chamber. A full-scale prototype detector has been built and successfully tested using a 1 MeV electron beam at the NIST Van de Graaff accelerator (see Refs. [10, 11]). The experimental results support the conclusions of a Monte Carlo simulation that the spectrometer will reduce the uncertainty in a due to electron backscatter to less than 0.5 %.

### 3.2 Electron Scatter From Collimators

Beta electrons that scatter from any material before hitting the detector will lose energy, leading to the same problem as detector backscatter. We have designed the collimators as thin tungsten annuli with chamfered knife edges. Computer simulations using the PENELOPE [12] electron-transport routine show that the fraction of scattered electrons reaching the detector is less than 0.05 % of the unscattered detected electrons.

### 3.3 Transverse Magnetic Fields

**Fig. 3:**
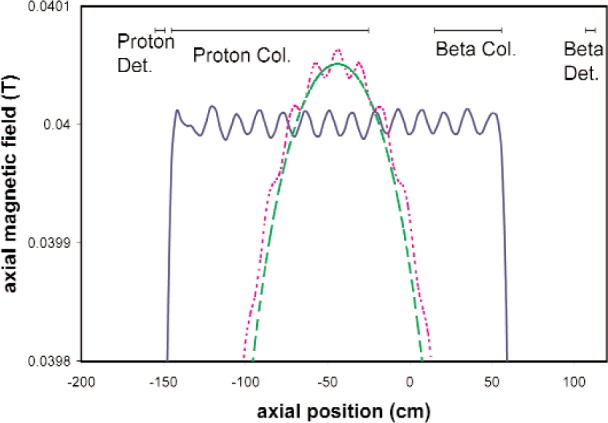
The axial magnetic field in the proton and electron transport regions using a single 5 m long solenoid (dashed line), a 5 m long array of equally spaced 7 cm long coils (dotted line), and a 2.6 m long array of unequally spaced 7 cm coils (solid line). We plan to use the latter configuration. The neutron beam is admitted at *z* = 0. Horizontal bars indicate the collimator regions, where uniformity is important, as well as the detector positions.

Transverse magnetic fields can lead to a false asymmetry. We must cancel background fields and create a highly uniform magnetic field while leaving an opening in the solenoid large enough to admit the neutron beam. An array of coils suitably spaced about 7 cm apart can distribute the error in the field in a rather uniform series of ripples (Fig. 3) whose effect tends to average away over the 1–2 helical turns of a proton trajectory. By carefully choosing the spacings and currents we can produce a field whose transverse components do not exceed 10^−4^ of the axial field. Thanks to the axial momentum boost from the accelerating electric field, the B field is too uniform to allow even the least favorable electrons to be trapped by the magnetic mirror effect and yields less than 10^−3^ false asymmetry in the proton populations.

### 3.4 Transverse Electric Fields

The electrostatic mirror must create a highly uniform axial electric field in the decay region so that we detect the same phase space for both proton populations. It must also let the protons and electrons pass freely though the field-generating electrodes and thus we must use wire grids. Moreover, the mirror region must be screened from the grounded vacuum chamber by a cylindrical grid or film, which will maintain a linear potential gradient at the circumference of the active volume. We have built finite-element models of the fields from such grids and run detailed Monte Carlo simulations of the complete electron-proton transport system. We found that grids of 20 µm diameter parallel wires on a 2 mm grid introduce a false asymmetry of (1.1 ± 0.5) 10^−4^.

### 3.5 Electron Detector Energy Resolution

To extract a from the data, the asymmetry must be easured as a function of electron energy and then divided by the electron speed to an accuracy of 0.5 % or better. This means that the centroid and shape of the electron detector response function must be known absolutely to within 1 %. This will be carefully studied and calibrated on a test beam.

### 3.6 Proton Detector Efficiency

The proton detector will consist of a thin, cooled silicon surface barrier detector and front-end preamp package surrounded by a hemispherical wire grid at −25 kV to accelerate the protons. We have successfully used a similar scheme to detect recoil protons in the past [13, 14]. At 25 keV the protons are wellseparated from the noise peak, and the thin detector is relatively insensitive to gamma and x-ray backgrounds. The electrostatic mirror ensures that all protons will have similar energies, although the angular distributions will be slightly different for the two groups. While this is not expected to be a serious issue, the equality of proton detector efficiency for the two groups must be verified by calculation and by a separate experiment using a low-energy proton source. If the detector efficiency is the same for both proton groups, backscatter from the proton detector itself simply removes events from the data without any systematic effect on *a*.

### 3.7 Beam-Related Background

Gamma and beta radiation created by neutron capture in the vicinity of the beam will cause backgrounds in the electron and proton detectors. This is a *delayed coincidence* experiment, so only accidental coincidences from background radiation will appear as background events in the data. The geometry of this experiment is advantageous for minimizing background. Both detectors are well-separated from the neutron beam and can be mostly surrounded by shielding. The areas of the apparatus closest to where the neutron beam passes will be covered with neutron absorbers containing enriched ^6^Li to absorb scattered neutrons without prompt gamma radiation. For this experiment we estimate a coincidence background rate of about 0.3 events per minute (signal/background of about 20), which can be removed for each energy slice using standard subtraction techniques.

## 4. Conclusions

We have presented a new method for measuring the electron-antineutrino correlation coefficient in free neutron decay that relies on the measurement of an asymmetry in the coincident detection of beta electrons and recoil protons. Unlike previous experiments, it does not require precise spectroscopy of low energy protons. The experiment will be built and tested at LENS and then run at NIST where a measurement of a to a precision of about 1 % is possible, five times smaller than the best previous experiment. A wide range of systematic effects has been considered, and we have shown that all can be controlled at the 0.5 % level.
